# Efficacy of Oral Intake of Hydrogen-Rich Jelly Intake on Gingival Inflammation: A Double-Blind, Placebo-Controlled and Exploratory Randomized Clinical Trial

**DOI:** 10.3390/healthcare13050577

**Published:** 2025-03-06

**Authors:** Takayuki Maruyama, Eiji Takayama, Shinichi Tokuno, Manabu Morita, Daisuke Ekuni

**Affiliations:** 1 Department of Preventive Dentistry, Faculty of Medicine, Dentistry and Pharmaceutical Sciences, Okayama University, Okayama 700-8558, Japan; dekuni7@md.okayama-u.ac.jp; 2 Advanced Research Center for Oral and Craniofacial Sciences, Okayama University Dental School, Okayama 700-8558, Japan; 3 Department of Oral Biochemistry, Asahi University School of Dentistry, Gifu 501-0296, Japan; takayama@dent.asahi-u.ac.jp; 4 Graduate School of Health Innovation, Kanagawa University of Human Services, Kawasaki 210-0821, Japan; s.tokuno-wm2@kuhs.ac.jp; 5 Department of Oral Health, Takarazuka University of Medical and Health Care, Takarazuka 666-0162, Japan; m.morita@tumh.ac.jp

**Keywords:** periodontal disease, oxidative stress, hydrogen, randomized controlled trial

## Abstract

**Background/Objectives**: Initiation and progression of periodontal disease include oxidative stress. Systemic application of antioxidants may provide clinical benefits against periodontal disease including gingivitis. Recently, a jelly containing a high concentration of hydrogen (40 ppm) was developed. We hypothesized that oral intake of this hydrogen-rich jelly may be safe and effective on gingivitis. This clinical trial was designed to investigate the safety and efficacy of oral intake of hydrogen-rich jelly against gingival inflammation. **Methods**: Participants with gingivitis were instructed to orally ingest 30 g of hydrogen-rich jelly (experimental group) or placebo jelly (control group) three times a day for 14 consecutive days. The primary outcome of this trial was the percentage of bleeding on probing (BOP) sites. Secondary outcomes were oral parameters, serum reactive oxygen metabolites, antioxidant capacity, oxidative index, concentrations of cytokine (interleukin [IL]-1β, IL-6, IL-10, IL-17, and tumor necrosis factor-alpha) in gingival crevicular fluid, and adverse events. For all parameters, Mann–Whitney *U* test was used for comparison between experimental and control groups. Analysis of covariance, controlling for baseline periodontal inflamed surface area, was performed to evaluate the association between the effect of the hydrogen-rich jelly and gingival inflammation. **Results**: In the experiment and control groups, the percentage of sites with BOP and PISA significantly decreased at the end of the experiment compared to the baseline. However, no significant differences were found between groups (*p* > 0.05). **Conclusions**: Administration of hydrogen-rich jelly for 14 days decreased gingival inflammation. However, no significant differences were identified compared to the control group.

## 1. Introduction

Periodontal disease is a chronic inflammatory disease of the periodontal tissues, and is characterized by gingival bleeding, periodontal pockets formation, inflammation of the periodontal ligament, destruction of the connective tissue, and the alveolar bone loss [1]. The initiation and progression of periodontal disease results from an imbalance between bacterial pathogens and immune responses [2]. These processes are mediated by oxidative stress. Reactive oxygen species (ROS) including superoxide, hydrogen peroxide, and hydroxyl anions are the products of immune responses to bacterial pathogens. However, increased ROS production or impaired antioxidant defenses result in a state of oxidative stress, causing oxidative damage to DNA, proteins, and lipids [3]. A relationship between periodontal disease and oxidative stress has been shown in previous studies [4,5,6,7]. Animal studies have reported that experimentally induced periodontitis induces the expression of 8-hydroxydeoxyguanosine (8-OHdG; an indicator of oxidative DNA damage) and nitrotyrosine (an indicator of oxidative protein damage) in fibroblasts or polymorphonuclear leukocytes [4,5]. In addition, experimental animal models of periodontitis have shown increased serum levels of ROS [6]. Our previous clinical study also demonstrated a positive association between serum ROS and bleeding on probing (BOP), and periodontal therapy (in the form of scaling and root planing) was effective in improving BOP and reducing plasma ROS for patients with chronic periodontitis [7]. BOP reflects inflammatory activity in the periodontal tissue and is a more sensitive indicator of periodontal inflammation than probing pocket depth (PPD) [8]. These studies indicate that periodontal inflammation may increase serum ROS and periodontal treatment may improve serum ROS by reducing inflammatory activity in periodontal tissues.

Systemic application of antioxidants may also provide clinical benefits against periodontal inflammation. A longitudinal study showed an inverse association between higher intakes of antioxidants, such as vitamin C, vitamin E, α-carotene, and β-carotene, and the number of teeth with periodontal disease progression were found in community-dwelling older Japanese [9]. Further, dietary intake of vegetables and fruits containing vitamin C, α-tocopherol, β-carotene, docosahexaenoic acid, and eicosapentaenoic acid were associated with reduced PPD after non-surgical periodontal treatment in non-smokers [10]. Reducing oxidative stress by ingesting antioxidants may thus be effective in improving periodontal disease.

Molecular hydrogen is considered to be a novel antioxidant that selectively reduces cytotoxic ROS [11]. In a previous study, intake of hydrogen-containing water (HW) suppressed ligature-induced periodontitis in rats by inhibiting increased serum ROS levels and lowering 8-OHdG and nitrotyrosine expression in periodontal tissue [12]. HW consumption also led to increased serum levels of total antioxidant capacity and improved clinical parameters among patients receiving non-surgical periodontal treatment [13]. These studies indicate that HW consumption might be useful for improving periodontal disease.

The maximum concentration of hydrogen dissolved in HW is about 1.6 ppm. A jelly containing a high concentration of hydrogen (40 ppm), approximately 25 times higher than that of HW, was recently developed. Consumption of this hydrogen-rich jelly is considered more effective than HW for ingesting molecular hydrogen and improving periodontal disease, especially gingivitis. However, the safety and efficacy of this hydrogen-rich jelly against gingivitis are unclear. We therefore designed this clinical study to investigate the safety and efficacy of oral intake of hydrogen-rich jelly against gingival inflammation among participants with gingivitis.

## 2. Materials and Methods

### 2.1. Trial Design

This single-center, parallel-design, double-blinded, placebo-controlled randomized clinical trial followed the Declaration of Helsinki and the Consolidated Standards of Reporting Trials (CONSORT) guidelines and checklist. This study was approved by the Okayama University Certified Review Board (approval no. CRB18-010) and was registered with the Japan Registry of Clinical Trials (no. jRCTs061180055). An independent dentist (M.M.) reviewed the safety data throughout the study. After the final statistical analysis, code breaking was performed. Eligibility criteria and outcomes did not change before and after the study.

### 2.2. Blinding

Participants, the periodontal examiner, the researchers measuring samples, and the researchers responsible for data analysis were all blinded to group allocations.

### 2.3. Participants

Participants were recruited among volunteers between February 2019 and March 2022 at the Department of Preventive Dentistry in Okayama University Hospital. Inclusion criteria were as follows: (1) participants with gingivitis with >20% of sites with BOP [14]; (2) age of 20–39 years; and (3) participants who provided written informed consent. Exclusion criteria were as follows: (1) participants with a medical history of antibacterial, anti-inflammatory, or antiallergic drugs within the preceding 2 weeks; (2) participants who regularly consumed HW; (3) participants with gelatin allergy; (4) participants with dysphagia; (5) participants with a history of smoking; (6) participants who were or might be pregnant; (7) breastfeeding participants; and (8) participants were judged to be inappropriate for study participation by the investigators for any other reason.

### 2.4. Randomization

Each participant was given a code number, and the coordinator (M.M.) randomly assigned the participants to one of two groups, i.e., the experimental group or the control group, using a computer-generated table. The allocation ratio was set at 1:1. The coordinators kept the sequentially numbered list in a sealed envelope.

### 2.5. Sample Size Calculation

Because this study was an exploratory trial, sample size calculation was not performed. However, we set the sample size and target number of cases at 10 per group, which was considered sufficient to assess the efficacy of the jelly based on the previous study [13].

### 2.6. Intervention

Participants were instructed to orally ingest 30 g of hydrogen-rich jelly (experimental group) or placebo jelly (control group) three times a day (after each meal) for 14 consecutive days. The hydrogen-rich jelly used in this study was manufactured by Shinryo Corporation (Kitakyushu, Fukuoka, Japan). The experimental jelly contains 40 ppm of hydrogen. Placebo jelly containing air instead of hydrogen was used as a control. Both jellies had the same taste, texture, and appearance of bubbles. The jelly was sealed in an aluminum film pouch, and a storage safety test demonstrated that no decrease in hydrogen content occurred with storage at 10–40 °C for 60 days.

### 2.7. Outcome Assessment

The primary outcome was the percentage of sites with BOP. Secondary outcomes included oral parameters, serum reactive oxygen metabolites (ROM) and antioxidant capacity (OXY-adsorbent test; OXY), cytokine concentrations in gingival crevicular fluid (GCF), and all adverse events.

One dentist (D.E.), who was blinded to the grouping, performed all oral examinations. PPD was measured at six sites per tooth (mesio-buccal, mid-buccal, disto-buccal, mesio-lingual, mid-lingual, and disto-lingual) for all teeth except the third molars using a color-coded probe (CP-11 Color-Coded Probe; Hu-Friedy, Chicago, IL, USA) [15]. The percentage of sites with BOP was calculated. The periodontal inflamed surface area (PISA) was calculated by the Excel spreadsheet program [16]. Oral hygiene status (O’Leary’s Plaque Control Record: PCR) was measured after erythrosine staining and was recorded at four sites in the cervical region (mesial, distal, buccal, and lingual) of each tooth [17]. The intra-examiner reliability of the PPD measurements, assessed by kappa statistics, was >0.8.

Blood was also obtained from the fingertip and centrifuged at 3000× *g* for 5 min to collect serum. The resulting serum sample was then used to determine serum ROM and OXY levels using the free radical evaluator (Free Radical Elective Evaluator; Diacron International, Grosseto, Italy) [18]. Measurements were performed according to the instructions from the manufacturer. ROM quantifies the total oxidative capacity of serum in response to N,N-diethyl-paraphenylenediamine in acidic buffer. Serum ROM levels are expressed in units of CARR U, where one CARR U is equivalent to 0.08 mg/dL of hydrogen peroxide. OXY quantifies the capacity of serum barrier against the strong oxidative action of a hypochlorous acid (HClO) solution. Serum OXY levels are expressed as micromolar concentrations of HClO consumed by 1 mL of serum (μmol HClO/mL). The balance between oxidative and antioxidant stress was calculated as an oxidative index. Because ROM and OXY have different units of measurement, to incorporate the parameters, standardized values of ROM and OXY were assessed using the following formula: sv-var = (v-var − m-var)/ds-var [19]. In this formula, sv-var represents the standard value of a certain parameter, v-var represents the original value, and m-var and ds-var represent the mean and standard deviation of the parameter, respectively. The oxidative index was calculated by subtracting the OXY standardized variable from the ROM standardized variable.

GCF samples were collected from the periodontal pocket on the buccal side of the anterior teeth. Before collecting the GCF samples, the selected sites were isolated with sterile cotton rolls and supragingival dental plaque was gently removed. The tooth was dried gently using an air syringe. After 2 min, GCF were collected by placing a membrane strip (2 × 6 mm, 0.22 μm pore size; Millipore, Burlington, MA, USA) at the gingival margin for 30 s. The strip was immediately transferred into a microtube containing 300 μL of 0.01 M phosphate-buffered saline (pH 7.2). Inflammatory cytokines (interleukin [IL]-1β, IL-6, IL-10, IL-17, and tumor necrosis factor [TNF]-α) in GCF were assessed using a multiplex fluorescent bead-based immunoassay and the Bio-Plex 200 Suspension Array System (Bio-Rad Laboratories, Hercules, CA, USA). Measurements were performed according to the instructions from the manufacturer. Detection limits ranged between 1 and 2.24 pg/mL, except for TNF-α, which had a detection threshold of 6.63 pg/mL.

Adverse events were assessed according to the Common Terminology Criteria for Adverse Events version 4.0 [20].

### 2.8. Statistical Analysis

Oral parameters including percentage of sites with BOP, serum ROM and OXY levels and oxidative index, and cytokines levels in GCF were compared between the experimental and control groups using the Mann–Whitney *U* test. A comparison of variables between the baseline and the end of the experiment in each group were performed using the Wilcoxon signed-rank test. Analysis of covariance, controlling for baseline PISA, was performed on changes between baseline and the end of the experiment to assess the association between effects of the hydrogen-rich jelly and gingival inflammation. Adjusted differences and 95% confidence intervals (CIs) were determined. All analyses were performed using intention-to-treat (ITT) analysis and SPSS 27.0J for Windows (IBM Japan, Tokyo, Japan). Values of *p* < 0.05 were considered to indicate statistical significance.

## 3. Results

**Figure 1:**
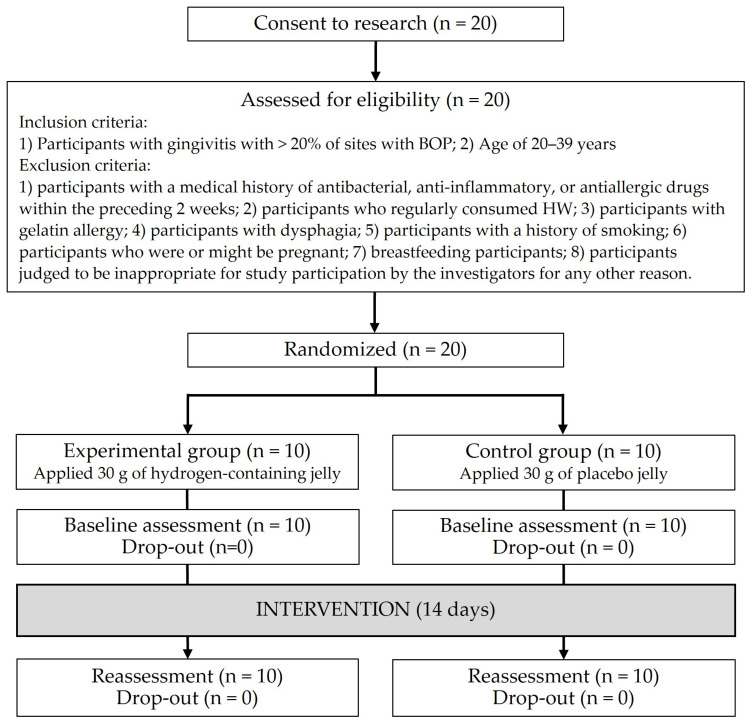
Flow chart.

Figure 1 shows the study flow chart. Twenty participants were enrolled, with no drop-outs (follow-up rate, 100%). The final ITT analysis was performed using the data from 20 participants.

The results for each parameter at baseline are shown in Table 1. Significant differences were observed in the percentage of sites with BOP and PISA between the experimental and control groups (*p* < 0.05).

The results for each parameter at the end of the experiment are shown in Table 2. There were no significant differences in all variables between the experimental and control groups (*p* > 0.05).

In both groups, the percentage of sites with BOP (*p* < 0.001) and PISA (*p* < 0.001) significantly decreased at the end of the experiment compared to the baseline. Other variables showed a tendency to decrease in both groups; however, no significant differences were observed (*p* > 0.05).

The changes between baseline and the end of the experiment for each parameter in the two groups are shown in Table 3. The adjusted differences (95%CI) in percentage of sites with BOP, mean PPD, PISA, and IL-17 in GCF showed negative values in the experimental and control groups. However, there were no statistically significant differences apparent between the two groups (*p* > 0.05). IL-10 and TNF-α levels in GCF showed a tendency to decrease in the experimental group. However, there were no statistically significant differences between the two groups (*p* > 0.05). Furthermore, there were no significant differences in other variables between the two groups (*p* > 0.05).

No adverse events or unintended effects were encountered during the trial. All participants completed each of the procedures without any trouble.

## 4. Discussion

To the best of our knowledge, this is the first study to examine the efficacy of high hydrogen concentration on gingival inflammation. Fourteen days after oral ingestion of hydrogen-rich jelly, the primary outcome (percentage of sites with BOP) decreased in the experimental group. However, no statistically significant differences were identified between groups. In our previous study [13], drinking HW improved the effects of non-surgical periodontal treatment in periodontitis patients and the percentage of sites with BOP was significantly decreased after 28 days. Although the design, hydrogen-containing materials, and targeted disease differed between this study and the previous study, the discrepancy suggests that hydrogen-containing materials had additional effects on periodontal inflammation in combination with non-surgical periodontal treatment or that the period (14 days) was short. Further studies are required.

In this study, the percentage of sites with BOP and PISA significantly decreased in both groups; however, no superiority was observed in the experimental group. Although participants were blinded to group allocations, Hawthorne effects might have occurred.

In terms of secondary outcomes, IL-17 and TNF-α levels in GCF were decreased in the experimental group; however, there were no statistically significant differences between the two groups. GCF contains biological markers for the immune-inflammatory response to dental plaque biofilm and downstream effects on the connective tissue attachment apparatus. This can reflect the periodontal status in realtime and can be used to detect indicators of bone metabolism, tissue remodeling, and inflammation [21]. IL-17 acts synergistically with TNF-α and IL-1 to induce the release of receptor activator of nuclear factor-kappa B ligand (RANKL), which binds to the receptor RANK on the surface of preosteoclasts, promoting osteoclastogenesis and initiating the process of bone destruction in periodontitis [22,23]. Therefore, oral ingestion of hydrogen-rich jelly might have the potential to prevent periodontal inflammation through reduction in IL-17 and TNF-α levels.

This study found that ingestion of hydrogen-rich jelly for 14 days tended to reduce serum oxidative stress and suppress increases in the oxidative index. However, there were no significant differences between the experimental and control groups. In the previous clinical study [13], participants drank HW for 56 days. Although serum oxidative stress was not significantly reduced after 14 days, serum oxidative stress was significantly reduced after 56 days [13]. These findings suggest that the effects of hydrogen-rich jelly on systemic oxidative stress are shown after more than 14 days.

Previous studies [12,24] have demonstrated that the application of hydrogen was beneficial for periodontal health independently. In a cell culture experiment, hydrogen water directly increased the migratory capacity of human gingival fibroblasts [24]. In an animal experiment [12], HW intake independently suppressed an increase in serum oxidative stress and reduced the expressions of 8-OHdG and nitrotyrosine in periodontal tissue. HW intake prevented the infiltration of polymorphonuclear leukocytes, osteoclast differentiation and the progression of periodontitis [12]. Although cell culture and animal experiments have demonstrated that hydrogen is effective for periodontal tissues, few clinical studies have demonstrated its efficacy. Large-scale and long-term clinical trials are needed to verify the efficacy of hydrogen on periodontal tissue.

Studies have shown that natural components may help maintain oral health. Chitosan has antibacterial and anti-inflammatory properties, and it may improve the management of oral diseases [25]. Also, propolis has antibacterial, anti-inflammatory, and antioxidant properties, and could effectively reduce gingival inflammation [26]. Natural components are safer than chemical components and can be used continuously for a long time.

This study had some limitations. First, all participants were recruited at Okayama University Hospital. This may limit the applicability of our findings to the general population. Second, all participants in this study had gingivitis. The effects of ingesting hydrogen-rich jelly on severe periodontitis may differ from our findings. Third, we could not investigate the additional effects of hydrogen-rich jelly in combination with periodontal treatment. Fourth, the period of the trial (14 days) was short. Finally, the sample size was small, with 10 participants per group. Larger clinical trials are needed to validate the results of this pilot study.

## 5. Conclusions

In the single-center, parallel-design, double-blinded, placebo-controlled randomized clinical trial, administration of hydrogen-rich jelly for 14 days decreased gingival inflammation (percentage of sites with BOP). However, no statistically significant differences were identified compared to the placebo control group. Larger and longer-term studies are needed to clarify the effect on gingival inflammation.

## Figures and Tables

**Table 1:** Characteristics of participants at baseline.

Variable	Experimental Group (n = 10)	Control Group (n = 10)
Age (year)	23 (21, 23) *	23 (21.5, 23)
Male	7 (70.0) ^†^	7 (70.0)
Percentage of sites with BOP (%)	37.65 (30.85, 45.95)	30.65 (26.125, 33.925)
Mean PPD (mm)	1.978 (1.872, 2.125)	1.942 (1.901, 2.005)
PISA (mm^2^)	422.45 (381.4, 537.725)	353.85 (294.7, 387.5)
PCR (%)	66.95 (56.25, 79.95)	62.05 (48.1, 78.0)
Serum ROM level (CARR U)	291.0 (267.75, 333.0)	295.0 (278.5, 394.25)
Serum OXY level (μmol HClO/mL)	400.0 (316.5, 417.475)	434.45 (330.4, 465.25)
Oxidative index	−0.248 (−1.053, 1.006)	0.019 (−1.022, 0.618)
IL-1β level (pg/mL)	34.40 (27.55, 42.22)	29.63 (16.09, 52.13)
IL-6 level (pg/mL)	1.36 (0.31, 12.33)	0.70 (0.47, 1.90)
IL-10 level (pg/mL)	3.39 (1.66, 6.50)	1.68 (0.83, 3.03)
IL-17 level (pg/mL)	2.21 (1.56, 3.43)	1.05 (0.47, 2.69)
TNF-α level (pg/mL)	110.64 (61.12, 133.22)	44.66 (21.40, 126.12)

* Median (interquartile range); ^†^ number (percentage).

**Table 2:** Characteristics of participants at the end of the experiment.

Variable	Experimental Group (n = 10)	Control Group (n = 10)	*p* Value
Percentage of sites with BOP (%)	25.9 (16.7, 30.025)	19.0 (15.625, 23.3)	0.123
Mean PPD (mm)	1.857 (1.765, 2.026)	1.849 (1.806, 1.886)	0.739
PISA (mm^2^)	292.05 (174.775, 340.775)	209.8 (173.85, 231.7)	0.075
PCR (%)	63.45 (47.95, 73.425)	58.05 (45.175, 75.925)	0.739
Serum ROM level (CARR U)	303.5 (271.0, 325.55)	321.0 (301.75, 369.5)	0.123
Serum OXY level (μmol HClO/mL)	377.15 (304.7, 438.15)	417.65 (336.35, 455.425)	0.393
Oxidative index	−0.188 (−0.910, 0.958)	0.615 (−0.690, 1.196)	0.393
IL-1β level (pg/mL)	25.53 (18.66, 31.32)	16.98 (6.16, 41.32)	0.529
IL-6 level (pg/mL)	1.59 (0.27, 2.51)	0.33 (0.27, 0.52)	0.075
IL-10 level (pg/mL)	1.59 (0.90, 4.69)	0.93 (0.35, 1.78)	0.243
IL-17 level (pg/mL)	1.50 (1.01, 2.69)	0.71 (0.50, 1.20)	0.113
TNF-α level (pg/mL)	50.38 (28.12, 111.32)	27.79 (22.43, 56.09)	0.356

Median (interquartile range). Mann–Whitney *U* test.

**Table 3:** Comparison of changes between baseline and end of the experiment.

Variable	Experimental Group (n = 10)	Control Group (n = 10)	*p* Value
Percentage of sites with BOP (%)	−13.85 (−18.9, −10.55)	−10.95 (−14.2, −6.6)	0.225
Mean PPD (mm)	−0.109 (−0.148, −0.088)	−0.11 (−0.151, −0.080)	0.116
PISA (mm^2^)	−167.05 (−241.43, −120.35)	−148.25 (−170.4, −109.275)	0.643
PCR (%)	−6.8 (−12.73, 12.73)	−1.35 (−11.6, 8.0)	0.868
Serum ROM level (CARR U)	−2.00 (−15.75, 14.50)	11.00 (−32.25, 41.5)	0.735
Serum OXY level (μmol HClO/mL)	−8.95 (−37.05, 41.075)	−4.750 (−45.33, 33.65)	0.650
Oxidative index	0.170 (−0.629, 0.418)	0.478 (−0.192, 0.905)	0.570
IL-1β level (pg/mL)	−7.81 (−20.25, 5.72)	−9.77 (−30.35, 1.75)	0.155
IL-6 level (pg/mL)	−1.78 (−11.10, 0.03)	−0.14 (−0.21, 0.01)	0.092
IL-10 level (pg/mL)	−1.11 (−3.21, −0.30)	−0.88 (−1.39, 0.46)	0.673
IL-17 level (pg/mL)	−0.79 (−1.20, −0.50)	−0.40 (−1.11, −0.05)	0.085
TNF-α level (pg/mL)	−37.05 (−83.79, −5.77)	−22.91 (−45.90, 7.92)	0.441

Adjusted difference (95% CI). Analysis of covariance (adjusted for baseline PISA as covariate).

## Data Availability

No new data were created or analyzed in this study. Data sharing is not applicable to this article.
